# Economic Burdens of Uncomplicated Malaria in Primary Health Care (PHC) Facilities of Plateau State, Nigeria: Patients’ Perspectives

**DOI:** 10.3390/ijerph20021093

**Published:** 2023-01-08

**Authors:** Nahlah Elkudssiah Ismail, Nanloh Samuel Jimam, Khang Wen Goh, Ching Siang Tan, Long Chiau Ming

**Affiliations:** 1 Malaysian Academy of Pharmacy, Puchong 47160, Malaysia; 2 Faculty of Pharmacy, MAHSA University, Jenjarom 42610, Malaysia; 3 Faculty of Pharmaceutical Sciences, University of Jos, Jos 930105, Nigeria; 4 Faculty of Data Science and Information Technology, INTI International University, Nilai 71800, Malaysia; 5 School of Pharmacy, KPJ Healthcare University College, Nilai 71800, Malaysia; 6 School of Medical and Life Sciences, Sunway University, Bandar Sunway 47500, Malaysia

**Keywords:** direct costs, human capital approach, indirect costs, primary health care facilities, uncomplicated malaria

## Abstract

**Highlights:**

**What are the main findings?**

- Malaria is an endemic disease throughout Nigeria, which is a developing country with high poverty levels of citizens, and patients’ inappropriate practices in combating malaria were reported as one of the causes.
- Material resources, health workers, and patient-related factors were implicated as reasons for such practices.

**What is the implication of the main finding?**

- This study will provide a comprehensive picture of the cost implications of treating the disease of patients and its projected economic impacts on the state.
- It might be useful in advising the health policymakers, as well as the healthcare professionals and patients, towards necessary attention and interventions.

**Abstract:**

Objectives: This study aims at evaluating the costs incurred by patients in Primary Healthcare facilities of Plateau State, Nigeria, due to uncomplicated malaria management. Methods: Patients’ information on resources used and absence from the labour market due to uncomplicated malaria illness were collected using the self-reported cost of illness instruments across 24 selected Primary Health Care (PHC) facilities in Plateau State. The collated data were used to estimate the direct medical and non-medical costs incurred by patients through the summation of the various costs paid out of pocket for the services; while the indirect cost was estimated using the human capital theory. All analyses were conducted through Microsoft Excel and IBM Statistical Package for Social Sciences (SPSS^®^) version 23 software. Results: The average direct cost per episode of uncomplicated malaria was estimated at NGN 2808.37/USD 7.39, while the indirect average money equivalence of the time lost due to the ailment was estimated at NGN 2717/USD 7.55, giving an average cost of treating uncomplicated malaria borne by patients in Plateau State per episode to be NGN 5525.37/USD 14.94. The projected annual cost of the disease was NGN 9, 921,671,307.22 (USD 27, 560,198.08). Conclusions: The study showed substantial financial costs borne by patients due to uncomplicated malaria in Plateau State, comprising 50.83% of direct cost and 49.17% of the indirect cost of medications.

## 1. Introduction

Malaria is a vector-borne disease caused by a parasitic protozoan of the genus *plasmodium* and is transmitted to humans through infected female mosquitoes known as *Anopheles gambiae* [1]. It is a worldwide public health problem, especially in Sub-Saharan Africa [2]. The impacts of the disease on populations include direct health impacts, in which the affected persons suffer from both physical and psychological pains, which may result in either morbidity or mortality; loss and/or decrease in economic activities of such patients and their caregivers, thereby leading to significant reductions in outputs [3,4,5]. Nigeria was reported as one of the countries that shared the largest burdens of the disease in Africa, with 25% of Africa’s burdens coming from the country [4,5,6].

Despite the huge amount of the country’s annual health budget for controlling the disease, in addition to various aids from external aids/development partners for controlling the disease, the benefits are still less felt by people, especially the rural dwellers, because it has not significantly reduced the disease incidence in the country [7]. This might be due to the reported correlations between malaria and poverty, whereby poverty sustains the conditions for malaria to thrive and malaria impedes economic growth, thereby keeping the population in poverty [8,9,10]. Lutala et al. [4] reported that people most affected by malaria are those living below the poverty line and Nigeria was shown to be one of the poorest countries in the world [6]. Due to the endemic nature of the disease in Nigeria, malaria affects the productivity levels of the people because such sick persons will not be able to work, or even if they can work, their inputs will be low compared to those of healthy persons; and there will also be time spent by both the patient and the caregivers during treatment, which cumulatively leads to a reduction in their earnings that would have been useful for the family needs. In addition, the little incomes earned by such populations are mostly spent on treating malaria-infected persons [11]. The combination of all these issues has a great impact on the patients and the country’s overall economic growth and productivity as a nation [3,5,12].

In order to ascertain the extent to which patients spent their resources during malaria management, there is every need to evaluate such management and services rendered in terms of their costs and consequences [13]. The cost of illness (COI) approach, also known as burdens of illness studies, is a partial economics evaluation method that is useful for the estimation of both the financial (direct and indirect) and intangible implications of the disease on the patients and society at large [13,14].

This includes the identification and estimation of various aspects of a disease’s impact, for example, money paid in consultation fees, laboratory tests, and cost of drugs or bed fees if admitted, and the non-healthcare direct cost including expenses incurred on transportation, food and others household expenditures related to the ailment [13,14]. Additionally included are the indirect costs, also known as productivity losses or productivity costs due to the ailment, which are borne by the individual patient or family, society, or employer [15], and can be best assessed using the human capital theory [16]. It is an important analytical method in the identification and estimation of the overall economic impact of ailments; with the advantage of defining the disease and its epidemiology in a particular population [17]. The analysis helps in knowing how much individuals/societies are spending on a particular disease and, by implication, the amount that would be saved if the disease were abolished.

Although some studies on the financial cost burdens of malaria on patients were conducted in some parts of Nigeria with varying outcomes, the majority of such studies were carried out in the South-Eastern region of the country [18,19,20,21,22]. The purpose of this study was to estimate the economic burdens of uncomplicated malaria management on patients receiving treatment in primary healthcare facilities of Plateau State, north-central Nigeria.

## 2. Methods

### 2.1. Study Design and Location

The prevalence-based bottom-up approach was used to assess the cost of uncomplicated malaria through patients’ perspectives in 24 PHC facilities across Plateau State, north-central Nigeria. The state has seventeen (17) Local Government Areas with a landmark of 30,913 km^2^ and population of 5,178,712 [23].

### 2.2. Sample Population, Size and Sampling Technique

The study was carried out on populations attending public PHC facilities across Plateau State for uncomplicated malaria management, including both genders and age groups.

Multistage probability sampling techniques including stratified and simple random approaches were used in the selection of participants. The sample size for the study was calculated in accordance with the World Health Organisation (WHO) manual on how to investigate drug use in health facilities [24,25]. Twenty-four (24) public PHC facilities were selected across the state and fifty (50) patients per PHC facility receiving treatment for uncomplicated malaria were recruited to participate in the study.

### 2.3. Ethical Approval

Approval for the study was granted by the Joint Research Review and Ethics Committee, Research Management Centre (RMC), MAHSA University, Malaysia (Ref. number: RMC/EC01/2016). Permission was also given by Plateau State Ministry of Health, Jos, Nigeria to access the PHC facilities, and the various directors of PHCs of the selected Local Government Areas (LGAs) were informed through memos prior to data collection.

### 2.4. Study Instruments

The COI instrument adapted from Salihu and Sanni [26] was used to estimate the cost impacts of uncomplicated malaria incurred by patients in PHC facilities. The instrument was divided into two subsections: subsection one consisted of relevant variables such as patients’ frequency of PHC facility visits for treatment, number of days spent on admission and cost (if admitted), means and cost of transportation, amount spent on registration, laboratory test, drugs and food per PHC visit, number of working hours when not sick and when sick, and national health insurance scheme (NHIS) registration status were used to estimate the cost of treating the disease using the patients’ perspectives approach.

### 2.5. Data Collection

Nine hundred and fifty-six (956) (79.67%) of the target populations receiving treatment for uncomplicated malaria who were available during the survey in the selected PHC facilities consented to participate in the study, who filled the self-reported instruments, and in situations where caregivers accompanied the patients, they were also given the instrument to fill for proper capturing of the cost incurred due to the ailment. The data were collected during the rainy season of Nigeria, between February and July 2017. Due to flooding and formation of water puddles that served as the breeding ground for mosquitoes, there was increased prevalence of malaria. To ensure that the correct information related to the various cost components paid by patients in accessing health services such as registration cost, laboratory and drug cost, and time spent waiting for medication among others were captured, the researcher only distributed the instrument to them to fill out at the point of drug dispensing, which was their last point of exit from the facility.

### 2.6. Economic Burden Analyses

The cost incurred by patients due to uncomplicated malaria in PHC facilities of Plateau State, Nigeria, was evaluated and presented.

- Direct costs

Descriptive statistics of all the related variables used in extracting information that were used in estimating the direct and indirect cost burdens of uncomplicated malaria on patients were presented as frequency and percentage distributions for categorical and continuous variables, in addition to the mean, standard deviations, and median and interquartile range (IQR) values for continuous variables. The direct and indirect cost burdens of the disease on patients per episode were estimated through the bottom-up approach, in naira (NGN) (Nigerian currency) and converted to United State Dollars (USD) based on 2017 exchange rate of NGN 360 to 1 USD when the study was conducted. All the data were managed and analysed through Microsoft Excel (version 2007) and the IBM-SPSS^®^) version 23 software.

The direct financial cost variables included patients registrations, laboratory diagnosis, and cost of drugs (medical costs) and costs of transportation, food, and accommodation if admitted (non-medical costs) since some of the patients were kept in the facilities under observation for some hours before discharged to continue with their medications at home.

In summary, the estimated direct financial cost of managing the disease was calculated as follows:

Direct cost (DC) = Medical cost (MC) + Non-medical cost (NMC)(1)

Medical cost (MC) = Patients registrations cost (PRC) + Laboratory diagnosis cost (LDC) + Cost of drugs (CoD)(2)

Non-medical cost (NMC) = Transportation cost (TC) + Feeding cost (FC) + Accommodation cost (AC)(3)

ii. Indirect costs (opportunity costs)

The indirect monetary cost (opportunity cost due to loss of productive time during the ailment) was estimated using human capital theory (HCT) approach [14,15]. HCT is a valuation method for the estimation of indirect costs of ailments. This approach values potential loss in productivity in terms of potential lost earnings, unlike the friction cost method, which values the loss in productivity over the length of time a particular organisation takes to return to its initial production and cost levels. The HCT approach is widely used in studying productivity losses due to illnesses or injuries of workers.

In the valuation of financial losses by patients due to malaria illness, the average time loss (days) due to the disease was first calculated, including time lost by caregivers for children and other patients who were taken to healthcare facilities by family members or friends, which was then followed by estimating its economic values using opportunity cost approach.

The opportunity cost (Z) due to uncomplicated malaria is the value of the output that is lost as a result of the sick person or caregiver’s inability to work either permanently or partially due to malaria-related morbidity and premature mortality; this was calculated based on Equation (4) [27,28].

Indirect cost (Z) = μ (Z1 + Z2 + Z3 … + Zn)(4)

where:

Z1 = waiting time at the PHC facilities;

Z2 = decreased in working time per day due to the ailment;

Z3 = time lost due to incapacitation;

Zn = other opportunity cost due to malaria ailment;

µ = market wages rate to represent the opportunity cost of lost time (for workers), or for self-employed and retired personnel, the average daily agricultural wage rate was used in the estimation of opportunity cost of lost time considering the large involvement of the study population in agriculture as main source of income.

### 2.7. Assumptions Made during the Calculations

The following assumptions were made during the calculations:

i. Government working hours start from 8 am to 4 pm (8 h) per day;
ii. Five working days per week, totalling 20 working days per month;
iii. A minimum wage of NGN 18,000.00 (naira) per month for salary earners = NGN 900 per day;
iv. Exchange rate: NGN 360.00 = 1 USD (based on Nigerian central bank foreign exchange rate of 2017);
v. Average minimum monthly income for farmers in Nigeria = ?

For the self-employed categories of patients, average daily agricultural wage rate was used in calculating their opportunity cost of lost time because majority of them were farmers. There were no fixed daily agriculture wages in Plateau State, the amount ranges from NGN 500 to NGN 1500 per person depending on the location, since the study covers the whole state, and an average of NGN 1000 was used as the daily agriculture wage rate for the non-salary earners, while a daily wage of NGN 900 based on NGN 18,000 minimum wage per month was used for the salary earners.

Therefore, average daily wage for patients


(5) $$=\frac{\mathrm{Average}\;\mathrm{daily}\;\mathrm{wage}\;\mathrm{for}\;\mathrm{salary}\;\mathrm{earners}\;+\;\mathrm{Average}\;\mathrm{daily}\;\mathrm{agriculture}\;\mathrm{wage}\;\mathrm{rate}\;\mathrm{for}\;\mathrm{non}\;-\;\mathrm{salary}\;\mathrm{earners}}{2}=\frac{900+1000}{2}=\frac{19000}{2}=\mathrm{NGN}950$$

Therefore, average daily wage of NGN 950 was used to estimate the average money equivalence of the time loss of patients due to uncomplicated malaria illness.

iii. Economic burden due to uncomplicated malaria on patients in Plateau State

Therefore, the total economic burdens on patients due to uncomplicated malaria treatment in PHC facilities of Plateau State was estimated by the summation of direct cost (medical and non-medical) and the indirect costs as follows:

The financial cost of illness = Direct costs + Indirect costs.

iv. Projected annual economic burden due to malaria on patients in Plateau State

In order to estimate the annual economic burden due to uncomplicated malari borne by patients in Plateau State, malaria prevalence rate baseline of 0.56 was used in the analysis. This was estimated from two sources: retrospective study aspect of the present study across PHC facilities of the state, which estimated the rate as 0.63 and similar prevalence studies conducted recently in Plateau State, which found the rate to be 0.4 [29]. The basis of using the average rate was to ensure wider coverage across the state since Nanvyat et al.’s [29] study was conducted only in the southern and northern parts of the state, while the present study covered the three zones (northern, central, and southern) of the state.

Based on the last census conducted in Nigeria, the population of Plateau State was 3,206,531 [30]. This was used when estimating the annual financial cost of malaria borne by patients in the state.

Projected economic burden = Mean cost × population × prevalence rate(6)

## 3. Results

A total of nine hundred and fifty-six (956) patients participated in the study and all of them attempted all the questions, which indicated their acceptability to participate in the survey. Five hundred and twenty-three (523) (54.7%) of them were females, while 433 (45.3%) were males. Four hundred and seventy-five (475) (49.69%) of the respondents were salary earners, while 481 (50.31%) were non-salary earners. Regarding their responses to the individual items, the majority of them (75.29%) had visited a PHC facility about 1–2 times for malaria-related ailments in the last 30 days, with the least (0.58%) indicating ≥ 5 times of visits to the facilities for the same purpose. Many respondents (50.73%) travelled to the PHC facilities by motorbike, popularly known as ‘going’, followed by 25.00% that preferred trekking and 10.46% that travelled by commercial cars. With a mean (±SD) of 3.12 (±3.90) hours’ time spent at PHC facilities by patients being attended to by healthcare workers per visit, most of them (42.68%) spent between 3–4 h, while 32.32% spent between 1.5 and 2.5 h, and only about 8.89% of the population spent ≥ 5 h in the PHC facilities, including those that were admitted whereby some spent not less than 48 h depending on the seriousness of their conditions. With the overall mean (±SD) working hours of 6.38 (± 3.21) per day before being sick, many worked for between 4 and 6 (46.23%) and 7 and 8 (29.39%) hours per day before they became sick but during post-diagnosis, the overall mean (±SD) daily working hours of the respondents reduced to 2.3 (±2.62), with many working for ≤3 (42.57%) and 4–6 h (37.34%) per day.

Apart from the indirect cost incurred by patients in terms of average time lost due to malaria ailment, various amounts of money (mean (±SD)) (in Naira) were spent by patients to gain access to treatment in PHC facilities. These included the cost of transportation to PHC facilities (NGN 178.28 (±214.20)), registration (NGN 138.34 (±426.78)), laboratory services (NGN 377.17 (±250.30)), purchase of anti-malarial drugs and co-medications (NGN 779.17 (±459.37)), food (NGN 168.55 (±270.34)), and accommodation (NGN 1166.86 (±1859.59)) for those who were admitted, which constituted the direct medical and non-medical cost burdens on the patients. About 4.08% of the study population were beneficiaries of health-related insurance policies/schemes. The details of payment distribution among the participants are presented in Table 1.

### 3.1. Direct Cost

#### 3.1.1. Direct Medical Cost for Patients

With a total registration cost of 93,932.86 Naira (NGN) (Nigeria currency) paid by the study population, 27.54% of them paid an average of NGN 138.34/USD 0.38 as a patient registration cost (PRC) before receiving treatment in the PHC facilities.

The average cost for laboratory services (blood microscopy and RDTs) paid by each patient amounted to NGN 377.17/USD 1.05. Similarly, the majority of patients (37.55%) paid an average of NGN 779.17/USD 2.16 per drug prescription, including antimalarial drugs and co-medications (Table 2).

#### 3.1.2. Direct Non-Medical Costs

Out of the 956 respondents that participated in the study, 59.2% of them use public transport as their means of visiting the PHC facilities for medication, and each of them spent approximately NGN 178.28/USD 0.5 on transportation. On the other hand, about 40.8% of the patients and their caregivers visited the PHC facilities through various means such as private cars, motorcycles and by trekking.

An average of NGN 168.55/USD 0.47 was spent on food by 33.98% of patients who cared to eat during the visit. Furthermore, the majority (93.18%) of the patients were treated as outpatients, with only a total of NGN 71,386/USD 198.1 spent on accommodation by the 6.82% of them that were admitted, at a mean total accommodation cost of NGN 1166.86/USD 2.83 naira per patient for the entire number of days admitted (Table 3).

#### 3.1.3. Total Direct Cost Incurred per Patient in Treating Uncomplicated Malaria

The average financial cost of treating uncomplicated malaria paid (direct medical and non-medical costs) by patients in the selected PHC facilities in Plateau State per episode was estimated to be NGN 2808.37/USD 7.39, the total cost of treating the disease among the study population per episode amounted to NGN 1,378,821.45/USD 3833.31. Out of this, 70.6% constituted direct medical costs, while 29.4% constituted the direct non-medical cost incurred by the patients during the management of the ailment (Table 4).

### 3.2. Indirect Costs of Uncomplicated Malaria on Patients

The average time lost by patients due to malaria ailment and the financial cost equivalence borne by the patients is presented in Table 5.

### 3.3. Economic Burden due to Uncomplicated Malaria on Patients in Plateau State

The average direct cost of treating uncomplicated malaria borne by patients in the selected PHC facilities in Plateau State per episode was estimated to be NGN 2808.37/USD 7.39, which was almost the same as the indirect cost due to the disease (NGN 2717/USD 7.55), as summarised in Table 6.

### 3.4. Projected Annual Economic Burden Due to Malaria on Patients in Plateau State

The total annual financial cost was estimated at NGN 9,921,671,307.22 (USD 27, 560,198.08), comprising 50.83% direct cost and 49.17% indirect cost borne by patients (Table 7).

## 4. Discussion

Cost of illness (COI) studies are an important analytical method for the identification and estimation of the overall economic impact of disease [17,31]. The present study followed the prevalence-based and ‘‘bottom up’’ approaches to estimate the financial cost impact of uncomplicated malaria disease in Plateau State, Nigeria. The outcome showed substantial financial burdens of the disease on the study population.

The average financial cost of treating uncomplicated malaria borne by patients in Plateau State per episode was estimated at NGN 5525.37/USD 15.35. In total, 50.81% (NGN 2808.37/USD 7.8) of the cost was due to the direct cost of the medications (direct medical and direct non-medical), which was slightly higher than the indirect cost due to the disease, which constituted about 49.19% (NGN 2717/USD 7.55) (Table 6). The result was in contrast to the outcome of a study conducted in a tertiary hospital in Benin [20], although, it was similar to the result of a household study carried out in Yenagoa metropolis (state capital of Bayelsa), which indicated that patients spent more on the direct cost of medications compared to the indirect cost components [32] but higher than similar studies conducted through the health system perspective in Enugu State, Nigeria [21]. It was similarly higher than the average cost of treating malaria episodes reported in Ghana [33] and south-central Ethiopia [34].

Detailed analyses on the average direct medical cost of the disease per episode indicated about 37.55% of the cost was due to drugs, followed by the cost of laboratory diagnostic tests (34.91%), and registration (27.54%) (Table 2). The observed poly-pharmacy practices in the management of the disease (Table 2) might be contributing factors to the high cost of drugs, although, the cost was lower than the average cost of USD 3.64 paid by patients in Enugu, south-eastern Nigeria for ACTs and co-medications per malaria episodes, as reported by Ezenduka et al. [35]. Furthermore, despite the fact that laboratory diagnoses were reported by Uzochukwu et al. [22] to be cost-effective, the observed amount paid for such services in the PHC facilities in the present study was considered high (Table 2), as most of the study populations were from rural settings who were living below the World Bank reported poverty line [36], and Omorogiuwa et al. [37] had earlier reported relationships between malaria and poverty. These cumulative costs of drugs and laboratory tests coupled with the high mean direct non-medical cost (Table 3) contributed to the high cost of medications in healthcare facilities (Table 4) and might be possible reasons why most patients preferred alternative means of accessing care when ill, especially patent medicine vendors where they are given drugs, mostly monotherapy, which is cheaper, without necessarily conducting laboratory investigations. This might have negative effects on the patients’ outcome of treatment, including resistance and treatment failure, with subsequent financial implications.

The projected estimated annual financial cost due to uncomplicated malaria borne by patients in Plateau State, based on a malaria prevalence rate of 0.56 was estimated as NGN 9,921,671,307.22 (USD 27,560,198.08) (Table 7), and this might have direct and indirect effects on the economy of the citizens and the state. A similar high cost of the disease was reported in other parts of Nigeria and some African countries [13].

## 5. Limitations

The use of minimum wage and agricultural wage rates in calculating daily wage equivalents for salary earners and non-salary earners could be over- or underestimated for some people, especially for the high salary earners, and this could imply fewer values of their time equivalent.

For respondents whose information was filled in by their caregivers due to their prevailing conditions, there could be some variations in information, especially regarding time spent at work and absenteeism; the amount paid for direct medical services was extracted from patients’ treatment cards and was not a problem since they were asked to fill the questionnaire after they had attended their last point of contact before exit (drug sections).

The resources involved in registration costs were mainly the opening of a folder for each patient for documentation of medication records and the registration cost paid by each patient before such folders were opened varies between NGN 20 to ≥NGN 1000 across the 24 selected PHC facilities. For the laboratory diagnosis, only malaria laboratory investigation was documented; patients’ paid between NGN 100 and NGN 2500, depending on the PHC facilities for such services to be rendered to each patient.

Though it may be an underestimation for those attending school, the time of absence from school could not be used since the main focus of the study was not on the impact of the disease on education.

Bias in findings due to the “hawthorne” effect was possible because some of the respondents might perform and answer better when they knew that their practices were under evaluation. Recall bias might also be a limiting factor to the study because when respondents were questioned about the resources consumed because of the illness, not all of them could exactly recall what they knew.

## 6. Conclusions

The study revealed a high burden of malaria disease on the Plateau State population, especially those living in the rural areas of the state, which might be causing significant financial hardships for them and, by extension, on the state economy. There is a need for urgent approaches to tackle this poverty-related ailment by ensuring that appropriate resources, both human and material-related, are provided in the rural health settings for case management of the disease, with regular awareness creation among both healthcare workers and the communities ensuring rational practices during medications, in addition to strategising toward poverty eradication.

## Figures and Tables

**Table 1:** Frequency distribution of patients’ cost variables for uncomplicated malaria (*N* = 956).

Variables	*n* (%)	Descriptive Statistics
**Number of visits to PHC facilities in past 30 days (times)**		
Mean (±SD)		2.53 (±1.93)
Median (IQR)		2 (0)
Minimum–Maximum		1–6
Have never	88 (9.23)	
1–2 times	720 (75.29)	
3–4 times	142 (14.90)	
≥5 times	6 (0.58)	
**Number of days on admission in PHC facility (mean (± SD))**		0.03 (±0.20)
<0.5	61 (6.38)	
0.5–1	9 (0.94)	
**Means of travel to PHC facilities**		
Trekking	239 (25.00)	
Bicycle	82 (8.58)	
Motorbike (Going)	485 (50.73)	
Commercial car	100 (10.46)	
Other means	50 (5.23)	
**Total time spent in PHC facilities per visit (hours)**		
Mean (±SD)		3.12 (±3.90)
Median (IQR)		3 (1)
Minimum–Maximum		0–48
≤1	154 (16.11)	
1.5–2.5	309 (32.32)	
3–4	408 (42.68)	
≥5	85 (8.89)	
**Transportation/visit cost to PHC facilities (NGN)**		
Mean (±SD)		178.28 (±214.2)
Median (IQR)		200 (200)
Minimum–Maximum		10–2000
≤NGN 100	266 (27.82)	
NGN150–250	272 (28.45)	
≥NGN 300	70 (7.32)	
**Registration cost before accessing PHC facilities (NGN)**		
Mean (±SD)		138.34 (±426.78)
Median (IQR)		50 (450)
Minimum–Maximum		20–1000
≤NGN 50	357 (37.34)	
NGN 60–NGN 200	236 (24.69)	
≥NGN 250	86 (9.00)	
**Blood taken for laboratory test**		
Yes	861 (90.06)	
No	95 (9.94)	
**Laboratory test cost (NGN)**		
Mean (±SD)		377.17 (±250.30)
Median (IQR)		350 (300)
Minimum–Maximum		100–2500
NGN 100	178 (18.62)	
NGN 150–350	291 (30.44)	
NGN 400–650	316 (33.05)	
≥NGN 700	76 (7.95)	
**Cost of prescribed drugs (NGN)**		
Mean (±SD)		779.17 (±459.37)
Median (IQR)		700 (1000)
Minimum–Maximum		80–5000
≤NGN 400	132 (13.81)	
NGN 450–800	483 (50.52)	
NGN 850–1250	200 (20.92)	
≥NGN 1300	111 (11.61)	
**Food cost during visit to PHC facilities (NGN)**		
Mean (±SD)		168.55 (±270.34)
Median (IQR)		100 (100)
Minimum–Maximum		20–900
≤NGN 100	210 (21.97)	
NGN 150–500	117 (12.24)	
≥NGN 550	22 (2.30)	
**Accommodation cost in PHC facilities (if admitted) (NGN)**		
Mean (±SD)		1166.86 (±1859.59)
Median (IQR)		500 (600)
Minimum–Maximum		10–10,000
≤NGN 500	36 (3.77)	
NGN 600–1000	19 (1.99)	
≥NGN 1200	15 (1.57)	
**Number of hours worked per day before sick (hours)**		
Mean (±SD)		6.38 (±3.21)
Median (IQR)		6 (6)
Minimum–Maximum		0–24
≤3	124 (12.97)	
4–6	442 (46.23)	
7–8	281 (29.39)	
>8	109 (11.40)	
**Number of hours worked per day when sick (hours)**		
Mean (±SD)		2.3 (±2.62)
Median (IQR)		3 (6)
Minimum–Maximum		2–12
≤3	407 (42.57)	
4–6	357 (37.34)	
7–8	102 (10.67)	
>8	90 (9.41)	
**Registered with any health-related insurance policy/scheme**		
Yes	39 (4.08)	
No	917 (95.92)	
**If registered, it covered**:		
Healthcare costs	16 (1.67)	
Income protection	20 (2.09)	
Any others	3 (0.31)	

**Table 2:** Average direct medical costs per case of uncomplicated malaria in Plateau State (in Naira (NGN) (Nigeria currency/USD) (*N* = 956).

			Total Costs for Study Population		
				Mean Cost	Standard Deviation
Variables	Frequency	Percentage	NGN/USD	NGN/USD	NGN/USD
					426.78/
Registration	679	27.54	93,932.86/260.92	138.34/0.38	1.19
Laboratory	861	34.91	324,743.37/902.06	377.17/1.05	250.3/0.7
					459.37/
Drugs	926	37.55	721,511.42/20,004.2	779.17/2.16	1.28
				1294.68/	
Total	2466	100	1,140,187.65/3167.18	3.59	323.89/0.9

**Table 3:** Average direct non-medical costs per case of uncomplicated malaria in Plateau State (in Naira) (NGN) (Nigeria currency/USD) (*N* = 956).

			Total Costs for Study Population		Standard
				Mean Cost	Deviation
Variables	Frequency	Percentage	NGN/USD	NGN/USD	NGN/USD
Transportation	608	59.2	108,406.4/304	178.28/0.5	214.2/0.6
				168.55/	270.34/
Food	349	33.98	58,841.4/164.03	0.47	0.75
				1166.86/	1859.59/
Accommodation	70	6.82	71,386/198.1	2.83	5.17
				1513.69/	573.59/
Total	1027	100	238,633.8/666.13	3.8	1.35

**Table 4:** Direct average and total costs per case of uncomplicated malaria on respondents in Plateau State (in Naira) (NGN) (Nigeria currency/USD) (*N* = 956).

			Total Costs for Study		
			Population		
				Mean Cost	Standard Deviation
Variables	Frequency	Percentage	NGN/USD	NGN/USD	NGN/USD
Direct					
medical cost	2466	70.6	1,140,187.65/3167.18	1294.68/3.59	323.89/0.9
Direct non-					
medical costs	1027	29.4	238,633.8/666.13	1513.69/3.8	573.59/1.35
Total	3493	100	1,378,821.45/3833.31	2808.37/7.39	418.51/1.03

**Table 5:** Average time lost (days) and money equivalence by patients due to uncomplicated malaria in Plateau State (in Naira) (NGN) (Nigeria currency/USD) (*N* = 956).

	Mean Time	Total Money	Mean Money	Standard
Variables	Lost (±SD)	Equivalence	Equivalence	Deviation
	Days	NGN/USD	NGN/USD	NGN/USD
Waiting time at PHC				
(days)	0.13 (±0.16)	118,066/327.96	123.5/0.34	152/0.42
Decrease in				
working time (days)	0.17 (±0.11)	154,394/428.87	161.5/0.45	104.5/0.29
Time lost due to				
incapacitation (days)	2.56 (±1.93)	2,324,992/6458.31	2432/6.76	1833.5/5.09
Total	2.86 (±1.39)	2,597,452/7215.14	2717/7.55	1320.5/3.67

**Table 6:** Economic burden per case of uncomplicated malaria per patient and study population in Plateau state (in Naira) (NGN) (Nigeria currency/USD) (*N* = 956).

Variables	Total Costs for Study Population	Means Cost	Standard Deviation
	NGN/USD	NGN/USD	NGN/USD
Direct cost	1,378,821.45/3833.31	2808.37/7.8	418.51/1.03
Indirect cost	2,597,452/7215.14	2717/7.55	1320.5/3.67
Total cost	3,976,273.45/11,048.45	5525.37/15.35	64.61/0.11

**Table 7:** Projected annual economic burden due to uncomplicated malaria on patients in Plateau State (in Naira) (NGN) (Nigeria currency/USD) (*N* = 956).

Variables	NGN	USD
Direct cost	5,042,870,260.1	14,007,972.94
Indirect cost	4,878,801,047.12	13,552,225.13
Total cost	9,921,671,307.22	27,560,198.07

## Data Availability

Data will be shared upon request and are subject to the applicable and relevant personal data protection laws and regulations.
